# Apocynin Prevents Cigarette Smoke-Induced Anxiety-Like Behavior and Preserves Microglial Profiles in Male Mice

**DOI:** 10.3390/antiox13070855

**Published:** 2024-07-16

**Authors:** Rana Alateeq, Alina Akhtar, Simone N. De Luca, Stanley M. H. Chan, Ross Vlahos

**Affiliations:** Respiratory Research Group, Centre for Respiratory Science and Health, School of Health and Biomedical Sciences, RMIT University, Bundoora, Melbourne, VIC 3083, Australia; s3729288@student.rmit.edu.au (R.A.); s3656686@student.rmit.edu.au (A.A.); simone.deluca@rmit.edu.au (S.N.D.L.);

**Keywords:** cigarette smoking, lung inflammation, anxiety, cognition, microglia, neurogenesis, oxidative stress

## Abstract

Chronic obstructive pulmonary disease (COPD) is the third leading cause of death globally and is primarily caused by cigarette smoking (CS). Neurocognitive comorbidities such as anxiety and cognitive impairments are common among people with COPD. CS-induced lung inflammation and oxidative stress may “spill-over” into the systemic circulation, driving the onset of these comorbidities. We investigated whether a prophylactic treatment with the NADPH Oxidase 2 (NOX2) inhibitor, apocynin, could prevent CS-induced neurocognitive impairments. Adult male BALB/c mice were exposed to CS (9 cigarettes/day, 5 days/week) or room air (sham) for 8 weeks with co-administration of apocynin (5 mg/kg, intraperitoneal injection once daily) or vehicle (0.01% DMSO in saline). Following 7 weeks of CS exposure, mice underwent behavioral testing to assess recognition and spatial memory (novel object recognition and Y maze, respectively) and anxiety-like behaviors (open field and elevated plus maze). Mice were then euthanized, and blood, lungs, and brains were collected. Apocynin partially improved CS-induced lung neutrophilia and reversed systemic inflammation (C-reactive protein) and oxidative stress (malondialdehyde). Apocynin exerted an anxiolytic effect in CS-exposed mice, which was associated with restored microglial profiles within the amygdala and hippocampus. Thus, targeting oxidative stress using apocynin can alleviate anxiety-like behaviors and could represent a novel strategy for managing COPD-related anxiety disorders.

## 1. Introduction

Chronic obstructive pulmonary disease (COPD) is the third leading cause of death globally and accounts for a substantial economic and social burden [1]. COPD represents a complex and debilitating respiratory condition characterized by persistent airflow limitation [1,2]. Beyond its well-established impact on the respiratory system, COPD is increasingly recognized as a condition with systemic consequences, leading to the development of extra-pulmonary comorbidities [3,4,5].

Cigarette smoking (CS) is the major cause of COPD in industrialized countries [1]. Over 4,700 known chemicals and free radicals have been identified in cigarettes, including reactive oxygen species (ROS) and reactive nitrogen species (RNS) [6]. Prolonged exposure to the noxious particles and gases leads to an inflammatory response, with the accumulation of activated alveolar macrophages and neutrophils driving persistent lung inflammation and oxidative stress [7,8]. Moreover, literature has shown that NADPH oxidase-2 (NOX2) is significantly elevated in the lungs in murine models of COPD and acute exacerbations of COPD [9,10]. We have previously shown that inhibition of NOX2 via treatment with apocynin partially resolves CS-induced lung inflammation by acting as a scavenger for superoxide (O_2_^−^) and other ROS molecules [3,4,10]. Inflammatory mediators and ROS/RNS activate immune cell sources, which may ‘spill-over’ into the systemic circulation, causing chronic low-grade systemic inflammation and oxidative stress, driving the manifestations of COPD comorbidities [11,12].

Individuals diagnosed with COPD commonly experience neuropsychological issues including anxiety, depression, and cognitive decline [11,13,14]. Clinical investigations suggest that anxiety affects approximately 35–68% of people with COPD, compared to 4% in the healthy population [15,16,17,18]. Moreover, studies indicate that cognitive impairments, including deficits in memory, attention, learning, and motor skills, afflict up to 61% of people with COPD [12,19,20]. Neurocognitive comorbidities are associated with increased mortality risk and diminished quality of life for people with COPD [21,22]. Additionally, a worsened disease prognosis is linked to increased rates of acute exacerbations induced by viral and bacterial infections, hospitalizations, and non-compliance with treatment [21,22]. Despite the high prevalence and complications, neurocognitive comorbidities in COPD often go undiagnosed and untreated [3,23], largely due to a limited understanding of the underlying mechanisms driving anxiety and cognitive impairments in COPD [12,13]. The current therapies available to manage COPD and its associated comorbidities are insufficient [24]. Current treatment strategies encompass both pharmacological approaches, such as corticosteroids and antidepressants, and non-pharmacological approaches, such as pulmonary rehabilitation [25]. However, such therapies have challenges and limitations, including potential side effects associated with corticosteroids and antidepressants and a lack of specific guidelines or tailored interventions targeting anxiety and cognitive impairment in COPD [24,25,26]. These limitations highlight the need for novel therapeutic strategies that target key mechanisms involved, such as oxidative stress, to effectively manage neurocognitive dysfunction in COPD.

Remodeling of the neuronal networks and architecture within key brain regions, including the amygdala, hippocampus, prefrontal cortex, and hypothalamus, is implicated in the pathogenicity of neurocognitive dysfunctions as well as the regulation of the stress response [27,28]. For example, atrophy of the hippocampus and amygdala in humans has been reported in neuropsychological disorders, including major depressive disorder and post-traumatic stress disorder [29,30]. A similar phenomenon was observed in rodents, with a decrease in neurogenesis within the hippocampus leading to cognitive deterioration and depressive-like and anxiety-like behaviors [31,32,33]. In the non-COPD setting, it has been proposed that chronic systemic inflammation and local neuroinflammation may result in changes in brain structure and synaptic plasticity, leading to anxiety and cognitive impairments [34,35]. Moreover, systemic inflammation and oxidative stress have been shown to be significant players in the pathogenesis of neurological comorbidities in COPD [36]. Individuals with COPD with symptoms of depression and anxiety have elevated systemic levels of inflammatory cytokines such as interleukin-6 (IL-6) and IL-1β [37,38,39]. Al-Shair et al. [40] suggested a relationship between tumor necrosis factor alpha (TNFα), depression, and fatigue scores in people with COPD. Another study found a reverse correlation between C-reactive protein (CRP) and fibrinogen levels and cognitive scores in individuals with COPD [41]. Moreover, a recent study and meta-analysis reported that high levels of DNA damage and malondialdehyde (MDA) were observed in people with COPD with cognitive impairment [42]. Nevertheless, the implicated neuroinflammatory mechanisms within specific brain regions in brain-related comorbidities remain to be explored in COPD.

One potential mechanism of CS-induced neurocognitive dysfunction in COPD is microglial-mediated neuroinflammation [43,44,45]. Microglia are the resident immune cells of the central nervous system (CNS) and actively regulate functions within the brain parenchyma, both in healthy and disease states [46]. Microglia are dynamic cells with highly motile processes that interact not only with the neurons [47], but also with other glial cells [48] and the vascular system [49]. In the physiological state, microglia extend their processes to actively survey the surrounding microenvironment, playing a crucial role in modulating and supporting neurogenesis by phagocytosing apoptotic cells, regulating neurogenesis levels influenced by inflammation and environmental enrichment, and actively modifying synaptic connections at the dendritic and synaptic levels in response to neuronal activity [12,50,51]. Upon stimulation, such as during injury or repair, microglia undergo morphological changes and secrete pro-inflammatory cytokines and oxidative stress mediators [12,50,51]. When this pro-inflammatory profile is uncontrolled or chronic, it results in impaired microglial function, which not only leads to excessive production of inflammatory and oxidative stress mediators but also induces altered modulation of neuronal activity, thereby driving impairments in neurocognition [12,51,52]. We have previously shown that exposure to CS for at least 8 weeks causes impairments in recognition memory and social recognition memory associated with altered microglial profiles within both the hippocampus and hypothalamus [3,12,53]. However, there are limited studies investigating the anxiety-like behavior in COPD and the underlying mechanisms responsible for this comorbidity. Thus, we aimed to investigate whether CS exposure induces an anxiety-like phenotype similar to humans with COPD and whether this is associated with altered neuroinflammatory profiles within key brain regions associated with anxiety. We also evaluated whether targeting the pro-inflammatory and oxidative stress profiles with apocynin, a NOX2 inhibitor [54,55], could prevent the CS-induced anxiety-like behavior and neuroinflammatory profiles.

## 2. Methods

### 2.1. Animals

Male BALB/c mice (7 weeks old) were obtained from the Animal Resource Centre Pty. Ltd. (Perth, WA, Australia) and housed in ambient laboratory conditions in the RMIT University animal research facility, with a 12-h light cycle (7 a.m. to 7 p.m.). All experiments were performed in accordance with the National Health and Medical Research Council’s (NHMRC) rules for animal experimentation and approved by the RMIT University Animal Ethics Committee (AEC1928).

### 2.2. Cigarette Smoke Exposure and Apocynin Treatment

**Figure 1:**
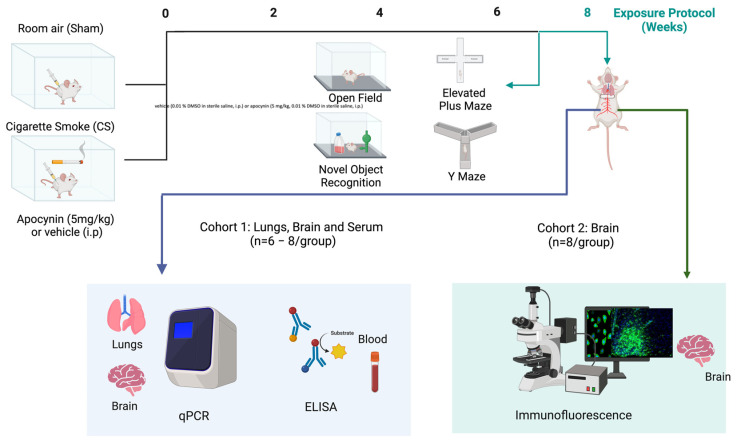
Timeline of the experimental design. Mice were either exposed to room air (sham) or cigarette smoke (CS; 9 cigarettes/day, 5 days/week) and administered either a vehicle (0.01% DMSO in sterile saline, i.p.) or apocynin (5 mg/kg, 0.01% DMSO in sterile saline, i.p.) for 8 weeks. A week before the protocol ended (week 7), neurocognitive assessments were conducted. Lungs were collected for qPCR, serum for ELISA, bronchoalveolar lavage fluid (BALF) for cytospins, and brains for qPCR, and immunohistochemistry.

Following an acclimatization period, mice were randomly assigned to either room air (sham) or CS groups with daily co-administration of either vehicle (saline containing ~0.01% dimethyl sulfoxide [DMSO]) or apocynin (5 mg/kg, dissolved in DMSO and diluted in sterile saline to reach a final concentration of 0.01% DMSO) administered via an intraperitoneal (i.p.) injection (n = 14 mice/group). Treatments were administered once daily, one hour prior to the initial CS exposure [3,4,10]. CS-exposed mice were exposed to 9 cigarettes per day, delivered over 3 sessions per day with a 2 h break between smoking sessions, for 5 days per week for 8 weeks, as previously published [3,4,53,56]. Sham animals were handled identically without CS exposure, as described in Figure 1.

### 2.3. Behavioral Assessment

Following 7 weeks of CS exposure, mice underwent cognitive and anxiety-like behavioral testing. All tests were completed between 8 a.m. and 1 p.m. to limit the potential effects of circadian rhythms on any of the parameters measured. To prevent the onset of a withdrawal phenotype, neurocognitive testing was performed between Tuesday and Friday. The mice were given at least 30 min to acclimate to the behavioral testing room before the commencement of each test, and all equipment was cleaned with 70% ethanol before and after use.

Open Field: To assess both locomotor activity and exploratory behaviors, mice underwent an open field task in a square black plywood arena [60 × 60 × 45 cm]. Mice were placed in the center of the arena and allowed to freely explore the maze for 8 min. The mouse navigated the arena by using visual clues of various geometric shapes and contrasting colors placed on each wall. To assess the behavioral outcomes, the mice were recorded with a camcorder positioned above the arena (Panasonic HC- W585M, Osaka, Japan) and later scored on EthoVision XT software (v11.5; Noldus Information Technology, Wageningen, The Netherlands). The arena was divided into 5 × 5 quadrants (each quadrant 12 cm long and 12 cm wide). The central zone was defined as the central 3 × 3 quadrant regions (36 × 36 cm) and the remaining area as the outer zone (edge). Mice were scored for total distance traveled (cm), total duration in the center (seconds [s]), and number of entries into the center. Representative heat maps were generated on EthoVision XT software [3].

Novel Object Recognition Task: Following habituation in the open field task, we examined hippocampal-dependent recognition memory in the novel object recognition task. Briefly, during the acquisition phase, mice were placed in the center of the arena and allowed to explore two identical objects equally spaced apart for 8 min. Upon completion of the acquisition phase, mice were returned to their home cage for a 1 h inter-trial interval (ITI). Following the ITI, mice were subjected to the same settings as the acquisition phase; however, one of the identical objects was replaced with a novel object. A Panasonic camcorder was used to record each session. Each video was manually scored to assess the interaction between the mouse and the objects. Interaction with the object was scored when the mouse sniffed or touched the object whilst standing on, sitting on, or leaning on the object, which did not result in scoring [3,12]. The preference index for novelty was determined by dividing the total time spent exploring the novel object by the total time spent interacting with both the novel and familiar objects multiplied by 100 (time _novel_/[time _novel_ + time _familiar_] × 100). The mouse could successfully perform the task if the novelty preference index was greater than 50%.

Spontaneous alternation in the Y-maze: To assess spatial working memory, mice were tested in the spontaneous alternation in the Y-maze task. The apparatus consisted of three symmetrical arms (30 cm long, 11 cm wide, and 17 cm high). Spatial cues (various geometric shapes and contrasting colors) were placed at the ends of the arms, allowing for spatial orientation. All sessions were recorded with an overhead webcam and assessed by a double-blinded investigator. Mice were placed at the end of one of the symmetrical arms and allowed to freely explore the maze for five minutes. The series of arm entries were recorded, and an entry was defined as all four limbs passing the central area connecting the arms. The number of sequential entries into each of the three arms as well as the number of arm entries were counted. The sequential entries into each of the three distinct arms during an overlapping triplet were referred to as alternations [3,12]. Data are presented as the percentage of spontaneous alternation (number of alternations/[total number of total arm entries − 2]) as well as the number of arm entries.

Elevated plus maze (EPM): To assess anxiety-like behavior, mice were tested in the EPM. The maze consisted of two open and two closed arms (50 cm long × 10 cm wide × 40 cm high walls of closed arms) raised 50 cm above the floor. Mice were individually placed in the center of the maze facing the open arm and allowed to freely explore the maze for 8 min. They were filmed with a webcam positioned above the arena and later manually scored using a code designed by the Python programming language (Python Software Foundation, Wilmington, DE, USA). Videos were scored for the number of entries in both closed and open arms and the duration in each arm (s). An arm entry was recorded when all 4 paws crossed the threshold of the arm [57].

### 2.4. Bronchoalveolar Lavage Fluid and Lung Collection

Mice were euthanized 90 min after the EPM behavioral task, between 9 a.m. and 11 a.m., with an overdose of sodium pentobarbital (240 mg/kg, i.p.; Virbac, Sydney, NSW, Australia). To assess lung inflammation, bronchoalveolar lavage fluid (BALF) was collected as previously described [12]. Briefly, a surgical tracheotomy was performed, and the lungs were lavaged in situ with 0.4 mL of chilled phosphate buffered saline (PBS), followed by three 0.3 mL aliquots of PBS, to generate approximately 1 mL of BALF per mouse. To determine the total number of viable immune cells, BALF was diluted with Acridine Orange/Ethidium Bromide (1:1; Invitrogen, Waltham, MA, USA), and the total number of viable cells was counted on a standard Neubauer hemocytometer under fluorescent light on an Olympus BX53 microscope (Olympus, Shinjuku City, Tokyo, Japan). Cytocentrifuge preparations (Shandon Cytospin 3, 400× *g*, 10 min) were performed on slides with approximately 5 × 10^4^ cells to differentiate the immune cell populations in the BALF. According to the manufacturer’s recommendations, dried cytospins were stained using the Shandon Kwik-Diff Kit^®^ (ThermoFisher Scientific, Waltham, MA, USA) and Merck’s Hemacolor (eosin and thiazine solutions; ThermoFisher Scientific) and mounted with Entellan (Merck, Melbourne, VIC, Australia). Differential cell counts were performed by a double-blinded investigator with at least 500 cells per slide counted and differentiated into macrophages, neutrophils, and lymphocytes using standard morphological criteria. Following BALF collection, whole lungs were perfused to clear blood via right ventricular perfusion with 10 mL of chilled PBS. Lungs were rapidly excised, snap frozen in liquid nitrogen, and stored at −80 °C until required for gene expression analysis.

### 2.5. Blood Collection

To assess systemic inflammation and oxidative stress, blood (approx. 700 μL) was collected via the inferior vena cava (approx. 16 h after the last CS exposure) in a Microvette 500 Serum Gel tube (Sarstedt, Hildesheim District, Lower Saxony, Germany) and left to clot for at least 15 min. Whole blood samples were centrifuged at 10,000× *g* for 5 min at room temperature (RT). Serum was then aspirated into fresh tubes and stored at −80 °C until required for measurement of systemic inflammation (CRP; RayBio Mouse CRP ELISA kit, RayBiotech Inc., Peachtree Corners, GA, USA) and oxidative stress (MDA; OxiSelect™TBARS Assay Kit, Cell Biolabs Inc., San Diego, CA, USA). The inter-assay variability for the CRP assay was <12% coefficient of variation (CV), the intra-assay was <10%, and the lower limit of detection was 150 pg/mL. All samples were measured in duplicate, and all treatment groups were assayed together according to the manufacturer’s instructions.

### 2.6. Brain Dissection

Following perfusion, brains were hemisected through the midline, and the right and left hemispheres were excised. The amygdalae, prefrontal cortex, and hippocampi from each hemisphere were dissected and stored at −80 °C until required for quantitative real-time PCR (RT-qPCR) (n = 6 mice/group). In a separate cohort, brains were excised and immersed in 4% paraformaldehyde in PBS (4 °C, pH 7.4) for 24 h, followed by cryoprotection in 20% sucrose (n = 8 mice/group), and stored for immunohistological analysis.

### 2.7. Quantitative Real-Time PCR

The lungs and key brain regions from the right hemisphere were collected as previously described [3,12], and HMC-3 cells (described below) were used to determine changes in candidate genes involved in pro-inflammatory and oxidative stress profiles. Total RNA from tissue and HMC-3 cells was isolated using a RNeasy^®^ Mini and a RNeasy^®^ Lipid Tissue Mini purification kit (Qiagen, Clayton, VIC, Australia), respectively. RNA was then transcribed to complementary cDNA by reverse transcriptase using a high capacity RNA-to-cDNA kit (ThermoFisher Scientific), following the manufacturer’s instructions. We performed qRT-PCR reactions using TaqMan assay reagents on the QuantStudio 7 Flex instrument (Applied Biosciences, Waltham, MA, USA) (Table 1). All reactions were performed in triplicates using *Gapdh* or *Rps18* as an internal housekeeping control for the lung and brain, respectively. We analyzed mRNA expression using the equation 2^ΔΔ*C*(^*^t^*^)^, where *C*(*t*) is the threshold cycle at which fluorescence is first detected significantly above background. Data are presented as fold expression relative to sham vehicle mice or control media for HMC-3 data.

### 2.8. Immunohistochemistry

To determine the effect of co-administration of CS and apocynin on neuropathology, brains were cut into 30 μM coronal sections in a one-in-five series using a cryostat (Leica Biosystems, Mount Waverley, VIC, Australia). To determine the microglial profile, we immunolabeled brain sections for Ionized Calcium-binding Adapter Molecule-1 (Iba-1), a marker of microglia. To assess neurogenesis, double immunofluorescence labeling was performed for mature (NeuN) and immature (Doublecortin [DCX]) neurons, while to examine synaptogenesis, we immunolabeled for the pre-synaptic marker synaptophysin (SYN). Briefly, free-floating sections were washed with tris-buffered saline with 0.1% Tween^®^ 20 Detergent (TBST) and blocked with 3% bovine serum albumin (BSA), 4% normal horse serum (NHS), and 0.3% Triton X-100 for 2 h at RT before incubation with the primary antibody overnight at 4 °C (Iba-1: 1:1000, anti-rabbit, [#019-19741]). RRID: AB_839504, Fujifilm Wako, Osaka, Japan. NeuN: 1:500, anti-rabbit, [#ab104225], RRID: AB_10711153, Abcam, Cambridge, UK. DCX: 1:1000, anti-guinea pig, [#ab2253], RRID: AB_1586992, Millipore, Burlington, MA, USA; Synaptophysin: 1:2000, anti-mouse, [#S5768], RRID: AB_477523, Sigma-Aldrich, Darmstadt, Germany). Sections were then transferred into the secondary antibody for 2 h at RT (Iba-1, NeuN: 1:500, Alexa Fluor 594 goat anti-rabbit, [#A-11012], RRID: AB_2534079, Invitrogen, Waltham, MA, USA. DCX: 1:500, Alexa Fluor 488 goat anti-guinea pig, [#A-11073], RRID: AB_2534117, Invitrogen, Waltham, MA, USA. Synaptophysin: 1:500, Alexa Fluor 488 rabbit anti-mouse, [#A-11001], RRID: AB_2534069, Invitrogen, Waltham, MA, USA), sections were mounted, and cover slipped with Fluoromount-G^TM^ with DAPI mounting medium (ThermoFisher Scientific, Waltham, MA, USA). Photomicrographs were taken at 20x magnification on the upright Olympus BX53 microscope (Olympus) or Olympus VS120 slide scanner (Olympus).

Microglia (Iba-1-positive cells) were quantified in four regions of the amygdala, including the lateral (LA), the basolateral (BLA), the basomedial (BMA), and the central nucleus (CeA), and in five regions of the hippocampus, including the CA1, CA3, hilus, subgranular (SG), and molecular regions of the dentate gyrus (DG). Mature neurons (NeuN) were quantified in the five regions of the hippocampus, including the CA1, CA3, hilus, SG, and molecular regions of the DG, while immature neurons (DCX) and synaptophysin were quantified in the SG and hilus regions of the DG, respectively. All brain regions were identified through the Franklin and Paxinos Mouse Brain Atlas, and three sections per brain (between 0.82 mm and 2.18 mm relative to the bregma for the amygdala and between 1.22 and 2.70 mm relative to the bregma for hippocampal sections) were analyzed. Microglia numbers were counted manually, and area per cell density was determined by calculating the object area fraction using Olympus cellSens Dimension™ (Olympus, Tokyo, Japan) software. NeuN and synaptophysin were analyzed using cellSens Dimension™ by thresholding the positive stains against the background. DCX-positive cells were manually counted in the DG at 40× magnification and were classified into one of three maturational stages: proliferative (no processes), intermediate (short or stumpy processes), or post-mitotic (long processes with branching into the granule cell layer and molecular layer) to assess the relative maturity of the immature neurons.

### 2.9. Cell Culture and Treatment

To explore whether pulmonary inflammation and oxidative stress directly affect microglial cells, we induced activation in human microglial cells (HMC-3) using conditioned media from human bronchial epithelial cells (BEAS-2B) exposed to cigarette smoke extract (CSE). Briefly, CSE was generated by bubbling the smoke of a single Winfield Red cigarette (Phillip Morris International, Melbourne, VIC, Australia) through 25 mL of pre-warmed DMEM (Dulbecco’s Modified Eagle Medium) at a rate of 5 mL·s^−1^, resulting in a 100% CSE stock solution [56]. The stock solution was then filtered and diluted in pre-warmed DMEM to achieve the required concentration (25% CSE). For condition medium experiments, human bronchial epithelial cells, BEAS-2B (CRL-9609; American Type Culture Collection, Manassas, VA, USA), were cultured in a monolayer in LHC-9 serum-free media (ThermoFisher Scientific) supplemented with 10% FBS and 1% penicillin/streptomycin in a humidified atmosphere (100% humidity, 5% CO_2_) at 37 °C. BEAS-2B cells were stimulated with 25% CSE for 6 h, and media was harvested, and the supernatant represents the CSE-indirect-conditioned media (CM). To assess if the NOX2 inhibitor, apocynin, could prevent microglial activation, apocynin was dissolved in DMSO to give a stock solution of 500 μM. The stock solution was diluted with pre-warmed DMEM to give a final concentration of 500 nM. BEAS-2B cells were treated with CM with apocynin for 6 h, and the supernatant was harvested to obtain the cotreatment (preventative) CM. In another experiment, BEAS-2B were treated with CM for 6 h, followed by treatment with apocynin for another 6 h, to obtain the therapeutic (therapy) CM.

The human microglial cells-3 (HMC-3) cell line was gifted to us by Professor Melissa Churchill, RMIT University. These experiments were performed using the HMC-3 cell line to determine the impact of cigarette smoke exposure and apocynin treatment on human microglia, as this is highly translatable to the clinic. HMC-3 cells provide a consistent and reproducible platform for experimental research, eliminating the variability seen with primary cells from donors. This consistency is vital for accurately assessing neuroinflammatory responses and the effects of therapeutic interventions. HMC-3 cells were cultured in a growth medium consisting of Minimum Essential Media (MEM; ThermoFisher Scientific) supplemented with 10% fetal bovine serum (FBS; ThermoFisher Scientific) and 1% penicillin/streptomycin (100 units mL penicillin and 100 μg mL streptomycin; ThermoFisher Scientific). Cells were cultured in a T-75 culture flask at a density of 2.1 × 10^6^ viable cells/cm^2^ and passaged at 70–80% confluence. Flasks were kept in a humidified incubator at 37 °C with 5% CO_2_. HMC-3 cells (n = 6 per treatment) were then incubated with either preventative or therapy CM for 24 h. Cells and supernatant were collected and stored at −80 °C for further analysis.

### 2.10. Western Blot

HMC-3 cells in six-well plates were collected and homogenized in 50 μL of RIPA lysis buffer supplemented with protease inhibitor cocktail and phosphate inhibitor cocktail (Sigma Aldrich, St. Louis, MO, USA). The samples were centrifuged at 14,000× *g* for 10 min at 4 °C, and the supernatant was collected. The total protein concentration was determined using a bicinchoninic acid assay as per manufacturer instructions (ThermoFisher Scientific). HMC-3 samples were diluted in diethyl pyrocarbonate (DEPC) water to achieve a final protein concentration of 10 μg.

Samples were heated at 95 °C for 10 min and denatured in SDS loading buffer (4× Laemmli buffer; 50 mM Tris-HCl pH 6.8, 2% SDS, 10% glycerol, 1% mercaptoethanol, 12.5 mM EDTA, 0.02% bromophenol blue). Samples were loaded into an SDS-PAGE gel (10%), separated by gel electrophoresis, and transferred onto a methanol-activated polyvinylidene difluoride membrane (Bio-Rad Laboratories Inc., Hercules, CA, USA) using the Trans-Blot Turbo system (Bio-Rad). Blots were blocked with 5% BSA in TBST at room temperature for 2 h. The blots were incubated with primary antibodies overnight at 4 °C (Phospho-SAPK/JNK (Thr183/Tyr185); 1:1000, anti-rabbit, [#9251], RRID: AB_3316, Cell Signaling Technology. SAPK/JNK; 1:1000, anti-rabbit, [#9252], RRID: AB_2250373, Cell Signaling Technology. Phospho-p44/42 MAPK (Erk1/2) (Thr202/Tyr204) (D13.14.4E) XP^®^; 1:1000, anti-rabbit, [#4370], RRID: AB_2315112, Cell Signaling Technology. p44/42 MAPK (Erk1/2); 1:1000, anti-rabbit, [#9102], RRID: AB_330744, Cell Signaling Technology. Phospho-p38 MAPK (Thr180/Tyr182) (3D7); 1:1000, anti-rabbit, [#9215], RRID: AB_331762, Cell Signaling Technology. p38 MAPK (D13E1) XP^®^; 1:1000, anti-rabbit, [#8690], RRID: AB_10999090, Cell Signaling Technology. The following day, membranes were incubated with HRP-conjugated secondary antibodies (anti-mouse IgG, HRP-linked Antibody, 1:1000, anti-mouse [#7076], RRID: AB_330924, Cell Signaling Technology, Danvers, MA, USA, or anti-rabbit IgG, HRP-linked Antibody, 1:2000, anti-rabbit [#7074], RRID: AB_2099233, Cell Signaling Technology) at RT for 1 h. The membranes were developed using Western Lighting Ultra Solution chemiluminescent reagents (Perkin Elmer, Waltham, MA, USA). β-actin was used as a standard for normalizing band intensities. Quantitative densitometry analysis of bands of interest was performed using Image Lab software, and representative blots are shown (Ver. 6.0, Bio-Rad).

### 2.11. Data Analysis

All in vivo data was analyzed using a two-way ANOVA as the dataset comprised of two independent variables—exposure (cigarette smoke or room air) and treatment (vehicle or apocynin), while in vitro data was analyzed using a one-way ANOVA as there was only one independent variable—treatment (cigarette smoke extract conditioned media and/or apocynin). Where significant interactions were found, we performed Tukey’s multiple comparison *post hoc* analysis. Statistical significance was assumed when *p* < 0.05. Data are presented as mean + SEM (standard error of the mean).

## 3. Results

### 3.1. Apocynin Treatment Reduces CS-Induced Neutrophilia in BALF

**Figure 2:**
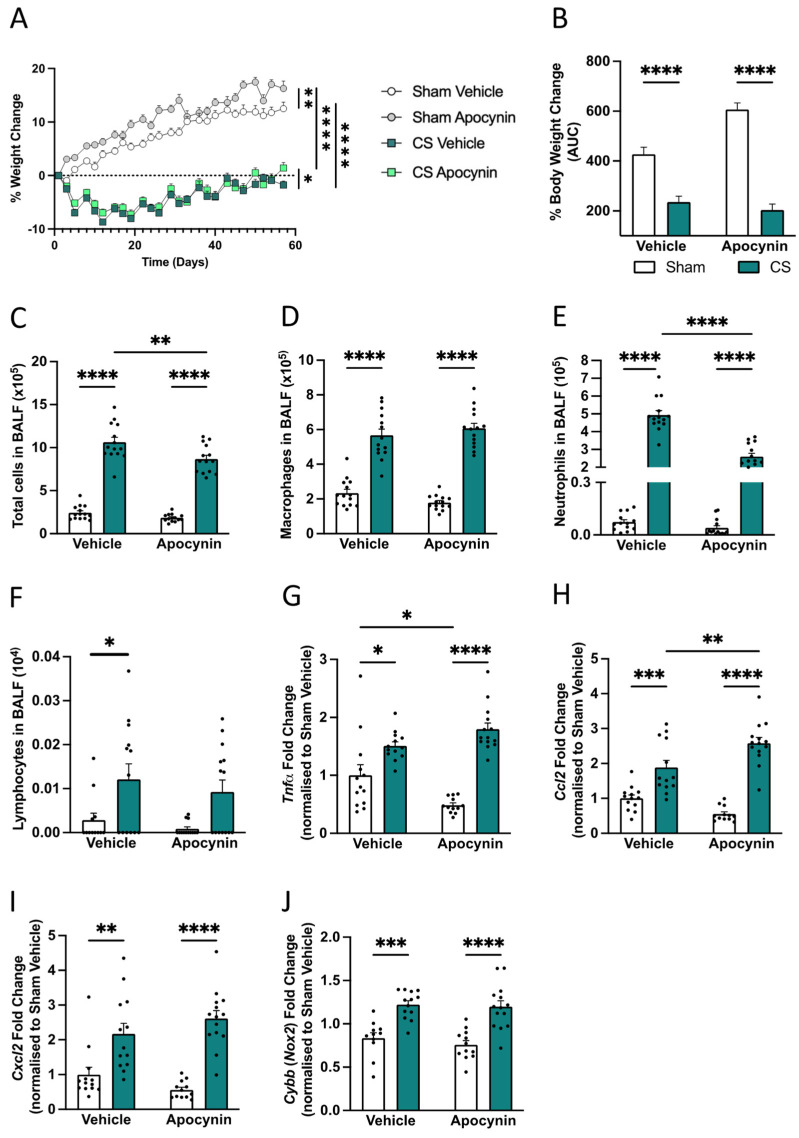
Effect of cigarette smoke (CS) exposure on bronchoalveolar lavage fluid (BALF) cellularity and inflammatory gene expression. Mice were exposed to CS or room air (sham) co-administered with apocynin/vehicle treatment for 8 weeks. (**A**) Body weight change (%; n = 14 mice/group). (**B**) Body weight change (% area under the curve [AUC]). (**C**) Total number of BALF cells (n = 14 mice/group). (**D**) Macrophages. (**E**) Neutrophils. (**F**) Lymphocytes. (**G**–**J**) Inflammatory and protease mRNA expression in the lung. (**G**) Tumor necrosis factor-alpha (*Tnfα*; n = 12–14 mice/group). (**H**) Chemokine ligand 2 (*Ccl2*). (**I**) Chemokine (C-X-C motif) ligand 2 (*Cxcl2*). (**J**) Cytochrome *b*-245, beta chain (*Cybb*). Data are expressed as mean + SEM. * *p* < 0.05, ** *p* < 0.01, *** *p* < 0.001, **** *p* < 0.0001.

Similar to our previous findings [56,58], exposure to CS for 8 weeks induced a significant reduction in weight gain when compared to sham vehicle mice (main effect of CS exposure: *F*_(1,51)_ = 229.0, *p* < 0.0001; Figure 2A,B). Sham apocynin mice gained significantly more weight compared to sham vehicle mice (main effect of treatment: *F*_(1,51)_ = 16.06, *p* = 0.0002); however, apocynin administration did not improve the area under the curve of percentage body weight change throughout the 8-week cigarette smoke exposure protocol. Interesting, similar to previous findings, apocynin treatment attenuated the weight loss at day 56 compared to CS vehicle mice (Figure 2A).

To examine whether a prophylactic apocynin treatment was effective in attenuating CS-induced immune cell recruitment to the lung, we performed differential cell count analysis on the BALF. Eight weeks of CS vehicle exposure led to an increase in the total number of immune cells within the BALF compared to sham vehicle mice (main effect of CS exposure: *F*_(1,52)_ = 394.5, *p* < 0.0001; Figure 2C), which was attributed to an increased number of macrophages (main effect of exposure: *F*_(1,52)_ = 207.5, *p* < 0.0001; Figure 2D), neutrophils (interaction of exposure by treatment: *F*_(1,52)_ = 53.96, *p* < 0.0001; Figure 2E), and lymphocytes (main effect of exposure: *F*_(1,52)_ = 15.87, *p* = 0.0002; Figure 2F). Consistent with our previously published data [56], pharmacological intervention using apocynin, significantly attenuated CS-induced BALF cellularity (main effect of apocynin treatment: *F*_(1,52)_ = 11.29, *p* = 0.0015; Figure 2C), which was attributed to a partial reduction in CS-induced neutrophilia (Figure 2E), but not macrophage nor lymphocyte recruitment (Figure 2D,F).

Furthermore, we evaluated the gene expression in whole lung tissue to characterize the inflammatory profile following apocynin administration. CS exposure significantly upregulated the expression of *Tnfα* (interaction between exposure *F*_(1,48)_ = 12.21, *p* = 0.0010; Figure 2G), *Ccl2* (interaction between exposure: *F*_(1,48)_= 15.06, *p* = 0.0003; Figure 2H)*, Cxcl2* (interaction of exposure by treatment: *F*_(1,48)_= 3.876, *p* = 0.0548; Figure 2), and *Nox2* (interaction between exposure: *F*_(1,48)_ = 1.885, *p* = 0.176; Figure 2J); however, prophylactic apocynin treatment was unable to prevent this pro-inflammatory profile.

### 3.2. Co-Administration of Apocynin-Attenuated CS-Induced Systemic Inflammation and Oxidative Stress

**Figure 3:**
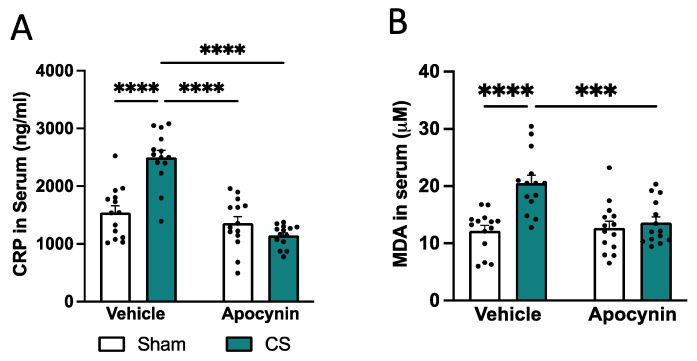
Apocynin reduced cigarette smoke (CS)-induced systemic inflammation and oxidative stress. Serum levels of (**A**) CRP and (**B**) MDA (n = 14 per group) were assessed using the ELISA and TBARS assay kits, respectively. Data are expressed as mean + SEM. *** *p* < 0.001, **** *p* < 0.0001.

To assess whether prophylactic administration of the Nox2 inhibitor apocynin was able to prevent systemic inflammation and oxidative stress, we measured serum CRP and lipid peroxidation levels (MDA), respectively. CS exposure caused a significant increase in serum CRP (Figure 3A) and lipid peroxidation (Figure 3B) compared to sham-vehicle mice. A prophylactic apocynin treatment prevented the elevated CRP (interaction of exposure by treatment: *F*_(1,52)_ = 29.72, *p* < 0.0001; Figure 3A) and MDA (interaction of exposure by treatment: *F*_(1,52)_ = 10.14, *p* = 0.0025; Figure 3B) levels following CS exposure similar to sham vehicle mice.

### 3.3. Apocynin-Alleviated CS-Induced Hyperlocomotion and Anxiety-Like Behavior

**Figure 4:**
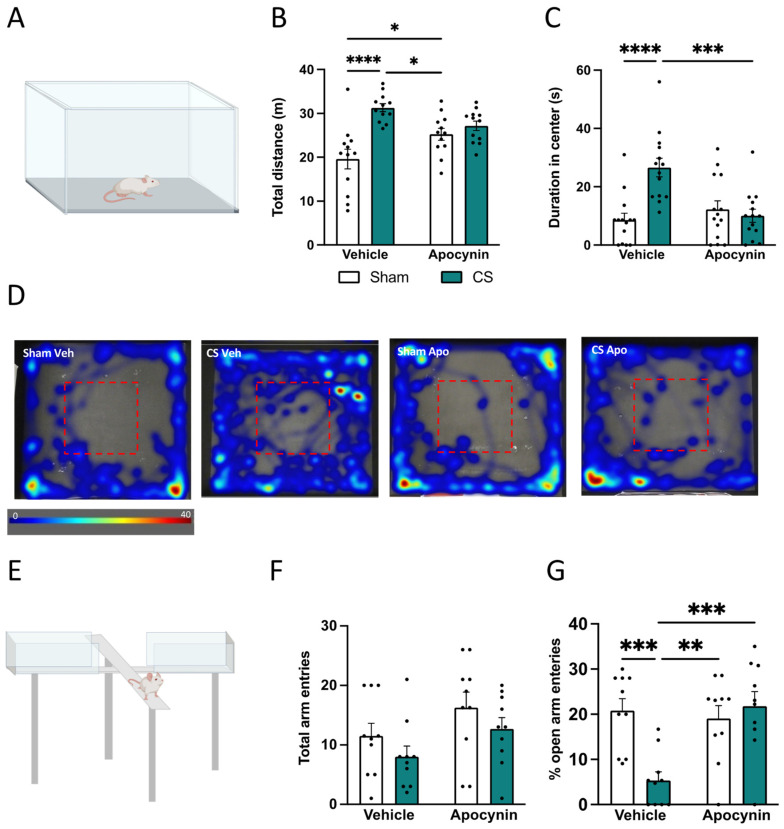
Apocynin alleviated cigarette smoke (CS)-induced hyperlocomotion and anxiety-like behavior. Behavioral testing was performed on mice one week prior to the protocol conclusion. (**A**) Schematic of an open field task. (**B**) Total distance (m) moved in the open field. (**C**) Time spent in the center of the open field arena (s; n = 12 per group) (**D**) Representative heat maps showcasing the time spent in the center (red box) of the open field arena. (**E**) Schematic of the elevated plus maze task. (**F**) Total arm entries. (**G**) Percentage (%) of open arm entries (n = 10 per group). Data are expressed as mean + SEM. * *p* < 0.05, ** *p* < 0.01, *** *p* < 0.001, **** *p* < 0.0001.

To investigate whether inhibition of NOX2 alleviated the CS-induced anxiety-like phenotype, we assessed the behavior in both the OF and EPM tasks. In the OF (Figure 4A), we observed increased locomotor activity in CS vehicle mice when compared to sham vehicle mice (interaction between exposure and treatment: *F*_(1,44)_ = 10.72, *p* = 0.0021; Figure 4B). This CS-induced hyperlocomotion was associated with a significant increase in center duration (interaction of exposure by treatment: *F*_(1,44)_ = 10.27, *p* = 0.0025; Figure 4C,D). Interestingly, CS exposure concomitant with apocynin administration decreased locomotor activity (Figure 4B) and center duration to that of sham vehicle mice (Figure 4C). In the EPM, CS exposure did not alter locomotor activity, with no differences in total arm entries (Figure 4E,F). CS-exposed vehicle mice displayed a significant reduction in the percentage of open arm entries when compared to sham mice, suggesting that CS exposure induced an anxiogenic phenotype in the EPM task. Mice treated prophylactically with apocynin showed an improvement in the percentage of open arm entries (interaction between exposure and treatment: *F*_(1,36)_ = 11.28, *p* = 0.0019; Figure 4G), thus showcasing that apocynin administration may be beneficial in preventing the anxiogenic phenotype induced by CS exposure.

### 3.4. Cigarette Smoke Impaired Recognition Memory but Not Spatial Working Memory

**Figure 5:**
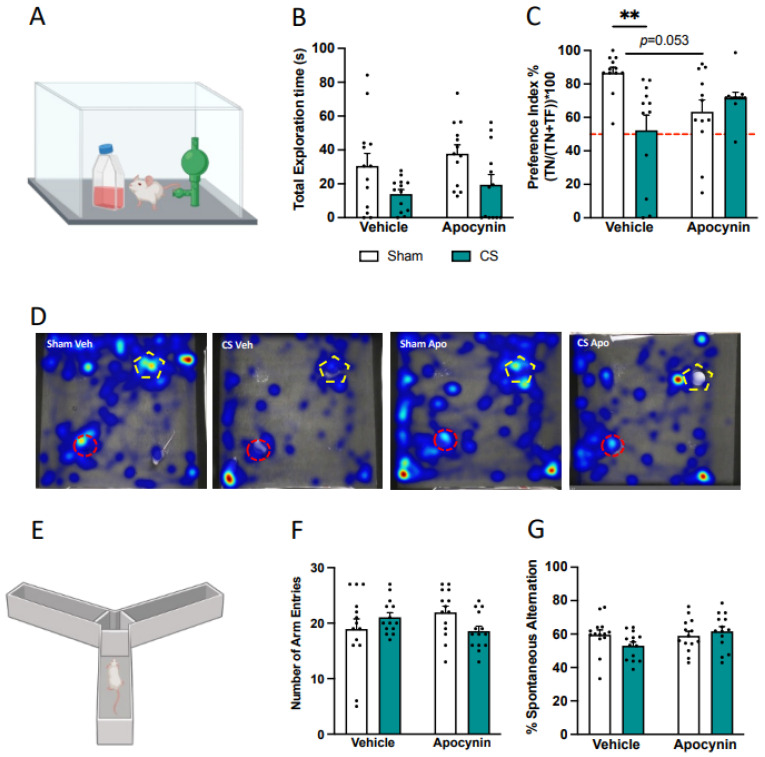
Cigarette smoke exposure impairs recognition memory. (**A**) Schematic of the Novel Object Recognition task with a familiar and novel object to determine novelty preference index. (**B**) Total time in seconds spent exploring objects during the acquisition phase. (**C**) Novelty Preference Index. (**D**) Representative heat maps of time spent with familiar (red circle) and novel (pentagon) objects. (**E**) Spontaneous alternation in the Y-maze test to assess spatial working memory. (**F**) Total number of arm entries. (**G**) Percentage spontaneous alternation in the Y-maze (n = 14 per group). Data are expressed as mean + SEM. ** *p* < 0.01.

We investigated both recognition and spatial working memory, as anxiety-like behavior is known to be linked to cognitive deficits. While CS exposure did not affect object exploration during the acquisition phase of the novel object recognition task (Figure 5A,B), mice exposed to CS were unable to distinguish between familiar and novel objects in the test phase. This indicates an impairment in recognition memory, and apocynin treatment did not reverse this deficit (*F*_(1,29)_ = 5.477, *p* = 0.026; Figure 5C,D). Interestingly, sham mice treated with apocynin showed a trend towards a decreased preference index for the novel object compared to vehicle-treated controls, although this did not reach significance (*p* = 0.053). CS exposure did not cause any spatial working memory impairments (Figure 5E–G).

### 3.5. Co-Administration of Cigarette Smoke and Apocynin Altered the Neuroinflammatory Profile in the Amygdala

**Figure 6:**
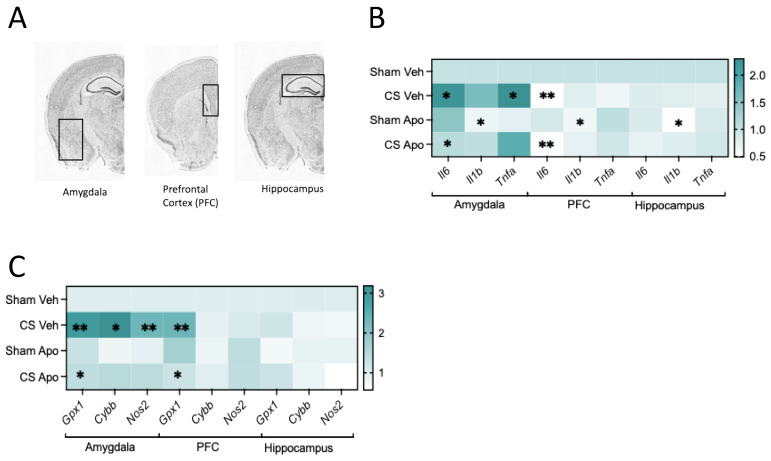
Apocynin altered the neuroinflammatory and oxidative stress profiles following cigarette smoke (CS) exposure. (**A**) Schematic of brain regions; amygdala, prefrontal cortex (PFC), and hippocampus. (**B**) Heat map of gene expression (n = 6 mice per group) of inflammatory markers including interleukin 6 (*Il6*), *Il1β,* and tumor necrosis factor-alpha (*Tnfα*). (**C**) Heat map of gene expression (n = 6 per group) of oxidative markers including glutathione peroxidase *(Gpx*), cytochrome b-245, beta chain (*Cybb*), and nitric oxide synthase 2 (*Nos2*). Data are expressed as means. * *p* < 0.05, ** *p* < 0.01.

Following our findings that CS exposure triggers pro-inflammatory and excessive oxidative stress in the lungs, along with anxiety-like behaviors, we investigated whether similar effects occur in key brain regions crucial for neurocognition, including the amygdala, hippocampus, and prefrontal cortex. We observed that CS exposure upregulated the mRNA expression of pro-inflammatory markers *Il6*, IL1 beta (*Il1β*), and *Tnf*α specifically within the amygdala, but, not in other regions (Figure 6A,B). Interestingly, apocynin treatment did not affect *Tnf*α expression but decreased the expression of both *Il6* and *Il1β* in the amygdala of CS-exposed mice similar to their sham counterparts (*Il6*: interaction of exposure by treatment: *F*_(1,20)_ = 8.644, *p* = 0.0081, *Il1b*: main effect of treatment: *F*_(1,20)_ = 4.749, *p* = 0.0414, and *Tnf*α: main effect of exposure: *F*_(1,20)_ = 12.77, *p* = 0.0019; Figure 6B).

Regardless of apocynin treatment, CS exposure significantly increased the expression of the oxidative stress genes *Nox2 (Cybb*) and nitric oxide synthase 2 (*Nos2*) in the amygdala (*Nox2*: main effect of exposure: *F*_(1,20)_ = 7.050, *p* = 0.0152, and *Nos2*: main effect of exposure: *F*_(1,20)_ = 13.10, *p* = 0.0017; Figure 6C). In contrast, CS exposure led to a significant increase in the antioxidant gene glutathione peroxidase (*Gpx*) in both the amygdala and prefrontal cortex regions. Interestingly, apocynin treatment was able to reverse this overexpression in both brain regions (amygdala: interaction of exposure by treatment: *F*_(1,20)_ = 8.470, *p* = 0.0087, prefrontal cortex: interaction of exposure by treatment: *F*_(1,20)_ = 17.31, *p* = 0.0005; Figure 6C).

### 3.6. Prophylactic Apocynin Preserves the Microglial Profile in the Amygdala and Hippocampus

**Figure 7:**
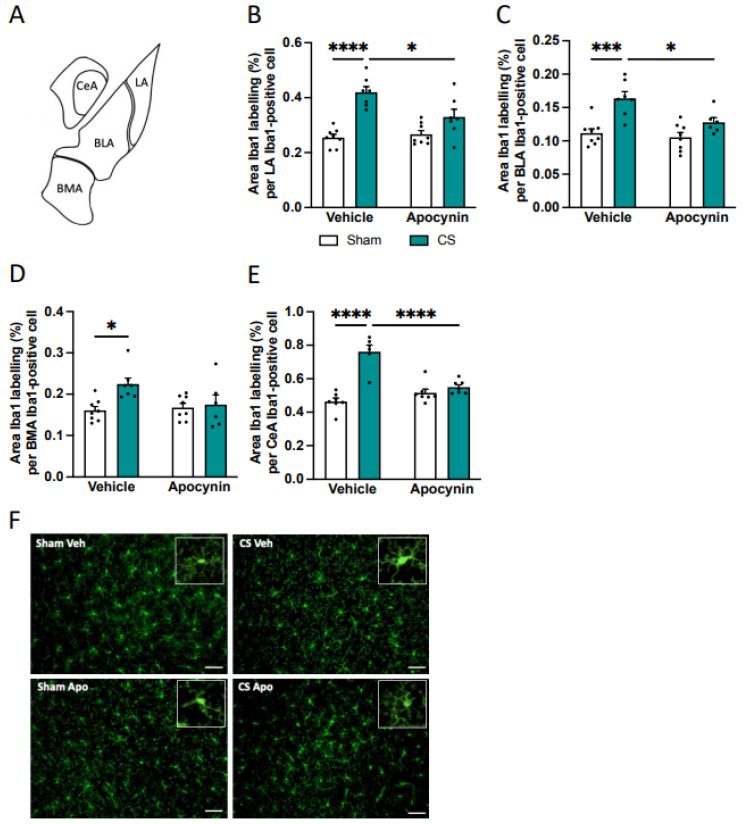
Apocynin treatment restored the microglial area per cell in the amygdala nuclei following cigarette smoke (CS) exposure. (**A**) Schematic of amygdala nuclei. (**B**–**E**) Area Iba-1 labeling per Iba-1 positive cell. (**B**) Lateral amygdala (LA; n = 8 per group). (**C**) Basolateral amygdala (BLA). (**D**) Basomedial amygdala (BMA). (**E**) Central nuclei amygdala (CeA). (**F**) Representative photomicrographs of the LA from room air (sham) and CS-exposed mice. White inlet represents a single magnified microglial cell. Scale bars are 20 μm. Data are expressed as mean + SEM. * *p* < 0.05, *** *p* < 0.001, **** *p* < 0.0001.

**Figure 8:**
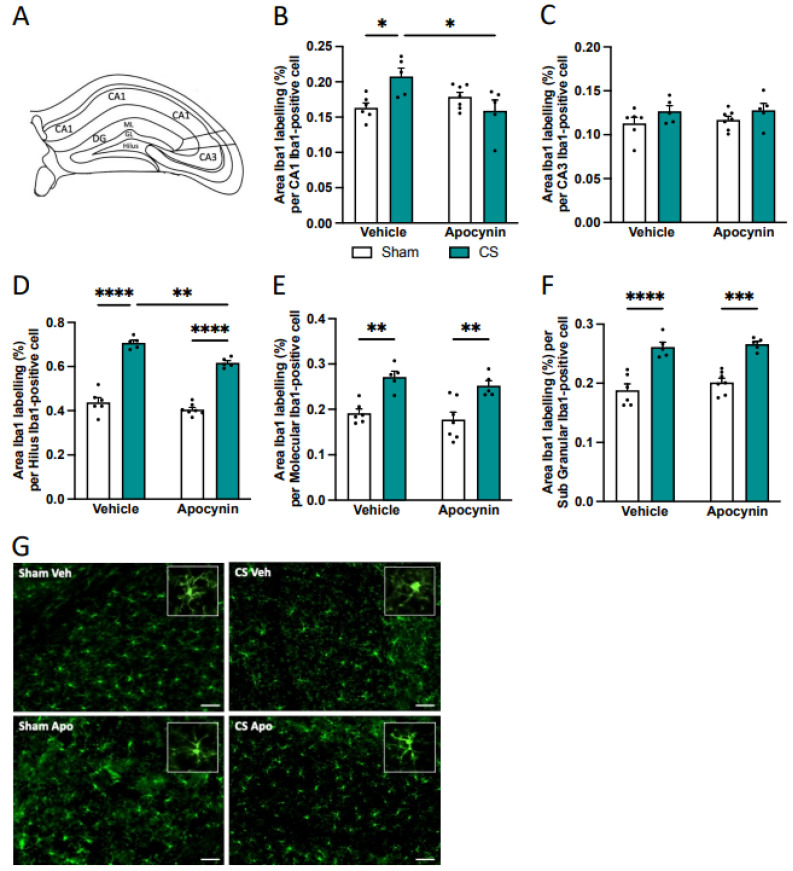
Apocynin restored the microglial area per cell in the hippocampus following cigarette smoke (CS) exposure. (**A**) Schematic of hippocampus subregions. (**B**–**F**) Area Iba-1 labeling per Iba-1 positive cell. (**B**) CA1. (**C**) CA3. (**D**) Hilus of the dentate gyrus (DG). (**E**) Molecular structure of the DG. (**F**) Sub-granular region of the DG (n = 6 per group). (**G**) Representative photomicrographs of the CA1 from room air (sham) and CS-exposed mice. White inlet represents a single magnified microglial cell. Scale bars are 20 μm. Data are expressed as mean + SEM. * *p* < 0.05, ** *p* < 0.01, *** *p* < 0.001, **** *p* < 0.0001.

Having established a link between CS-induced anxiety, cognitive dysfunction, and neuroinflammatory gene expression in brain regions crucial for neurocognition, we next investigated whether CS exposure alters the microglial number and morphology within the amygdala (Figure 7A) and hippocampus (Figure 8A), areas known to be involved in these processes. CS exposure significantly decreased the number of Iba-1-positive cells in the LA and CeA nuclei of the amygdala compared to sham vehicle, and this was prevented by the prophylactic administration of apocynin in the CeA nucleus only (LA: main effect of exposure: *F*_(1,26)_ = 24.04, *p* < 0.0001, BLA: main effect of exposure: *F*_(1,25)_ = 11.80, *p* = 0.0021, BMA: main effect of exposure: *F*_(1,25)_ = 6.212, *p* = 0.0197, CeA: interaction of exposure by treatment: *F*_(1,23)_ = 5.090, *p* = 0.0339; Table 2). Moreover, CS exposure led to an altered microglial morphological profile in the amygdala as evident by an increased Iba-1 positive area per cell within all the amygdala nuclei. Concomitant apocynin therapy was able to prevent the increased area per cell induced by CS exposure to sham vehicle mice in all nuclei of the amygdala except the BMA (LA: interaction of exposure by treatment: *F*_(1,26)_ = 7.351, *p* = 0.0117; Figure 7B,F; BLA: main effect of treatment: *F*_(1,25)_ = 7.096, *p* = 0.0133; Figure 7C; BMA: main effect of exposure: *F*_(1,25)_ = 6.196, *p* = 0.0198; Figure 7D; CeA: interaction of exposure by treatment: *F*_(1,23)_ = 27.72, *p* < 0.0001; Figure 7E).

Within the hippocampus, CS exposure caused a significant reduction in the number of Iba-1-positive cells within the molecular region of the dentate gyrus compared to sham mice; however, unlike the amygdala, prophylactic administration of apocynin was unable to prevent the reduced number of microglial cells (main effect of exposure: *F*_(1,19)_ = 8.034, *p* = 0.0106; Table 3). We found no differences in the number of Iba-1-positive cells in other regions of the hippocampus (Table 3). Similar to our findings in the amygdala, microglia from vehicle-treated mice exposed to CS showed an increased area per cell in all hippocampal subregions except the CA3 (Figure 8). Apocynin administration completely prevented the increase in microglial area per cell observed in the CA1 region of CS-exposed mice, restoring their microglial profile back to sham levels (interaction of exposure by treatment: *F*_(1,19)_ = 10.44, *p* = 0.0044; Figure 8B,G). Prophylactic apocynin treatment partially restored the microglial area per cell within the hilus region of the dentate gyrus, but not to sham levels (main effect of treatment: *F*_(1,19)_ = 18.04, *p* = 0.0004; Figure 8D). Apocynin did not alter the microglia profile within the molecular and sub-granular (SG) regions of the dentate gyrus (molecular: main effect of exposure: *F*_(1,19)_ = 32.47, *p* < 0.0001; Figure 8E; SG: main effect of exposure: *F*_(1,19)_ = 68.34, *p* < 0.0001; Figure 8F).

### 3.7. Cigarette Smoking Altered the Hippocampal Neuronal Profile

**Figure 9:**
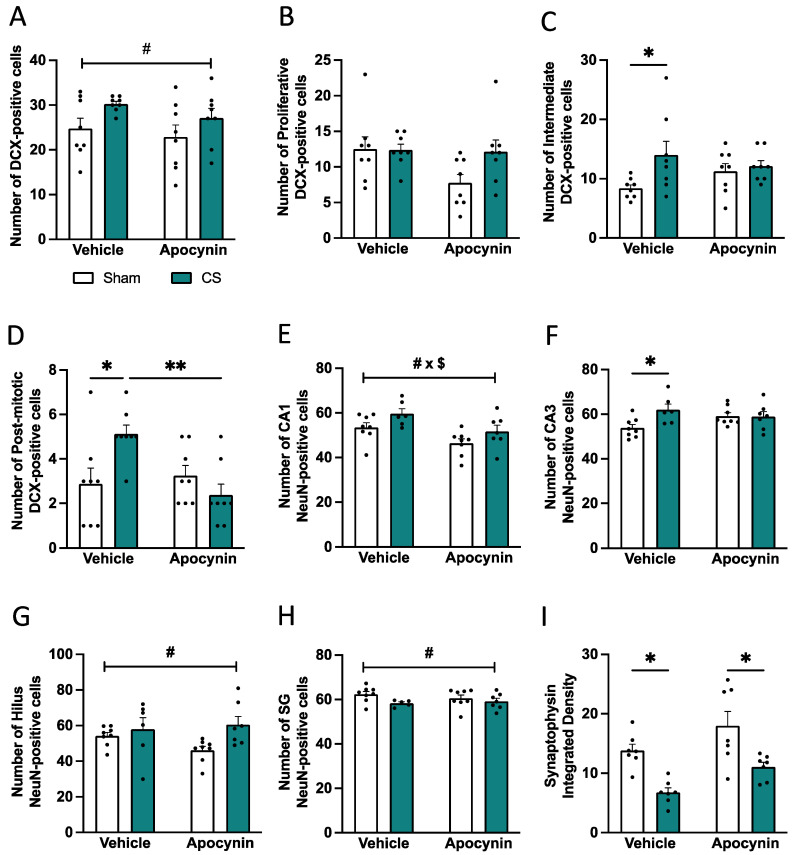
Cigarette smoke (CS) exposure alters hippocampal neurogenesis. (**A**) Number of immature neurons (doublecortin [DCX]). (**B**) Number of proliferative DCX-positive cells. (**C**) Number of intermediate DCX-positive cells. (**D**) Number of post-mitotic DCX-positive cells. (**E**–**H**) Number of mature neurons (NeuN) in the hippocampus; (**I**) Density of synaptophysin in the hilus region of the dentate gyrus (n = 8 per group). Data are expressed as mean + SEM. * *p* < 0.05, ** *p* < 0.01. # indicates a main effect of CS exposure. # x $ interaction between exposure and treatment.

While microglia in healthy rodents support neurogenesis and synaptogenesis by regulating immature and mature dendritic spines for intact cognition, chronic microglial activation can be detrimental, leading to neuronal loss and altered synaptic engulfment, ultimately resulting in neurocognitive dysfunction. Thus, we sought to investigate the hippocampal neuronal profile following 8 weeks of CS exposure concurrent with apocynin administration. CS mice had an increase in the number of DCX-positive cells (immature neurons) in the SG region of the dentate gyrus compared to sham vehicle mice (*F*_(1,22)_ = 6.669; *p =* 0.0170; Figure 9A). Although CS exposure did not impact the number of proliferative DCX-positive cells (Figure 9B), CS exposure induced a significant increase in the number of intermediate (Figure 9C) and post-mitotic DCX-positive cells (Figure 9D) compared to sham-vehicle mice. Prophylactic apocynin administration was able to prevent the increased number of post-mitotic DCX-positive cells (interaction between exposure and treatment: *F*_(1,21)_ = 6.297; *p* = 0.0204; Figure 9D). NeuN, a marker of mature neurons, was increased following exposure to CS in the hippocampus compared to sham mice, and apocynin did not restore this profile (CA1: *F*_(1,25)_ = 6.205; *p* = 0.0197; Figure 9E; CA3: *F*_(1,25)_ = 4.729; *p* = 0.0393; Figure 9F; hilus: *F*_(1,26)_ = 7.104; *p* = 0.0130; Figure 9G; SG: *F*_(1,26)_ = 7.028; *p =* 0.0135; Figure 9H). CS-exposed mice displayed a significant reduction in the pre-synaptic marker, synaptophysin, within the hilus region of the dentate gyrus, and apocynin was unable to restore the synaptic density (*F*_(1,24)_ = 23.62; *p* < 0.0001; Figure 9I).

### 3.8. Treatment with Apocynin Prevents CSE-Induced Inflammation and Oxidative Stress In Vitro in Human Microglial Cells

**Figure 10:**
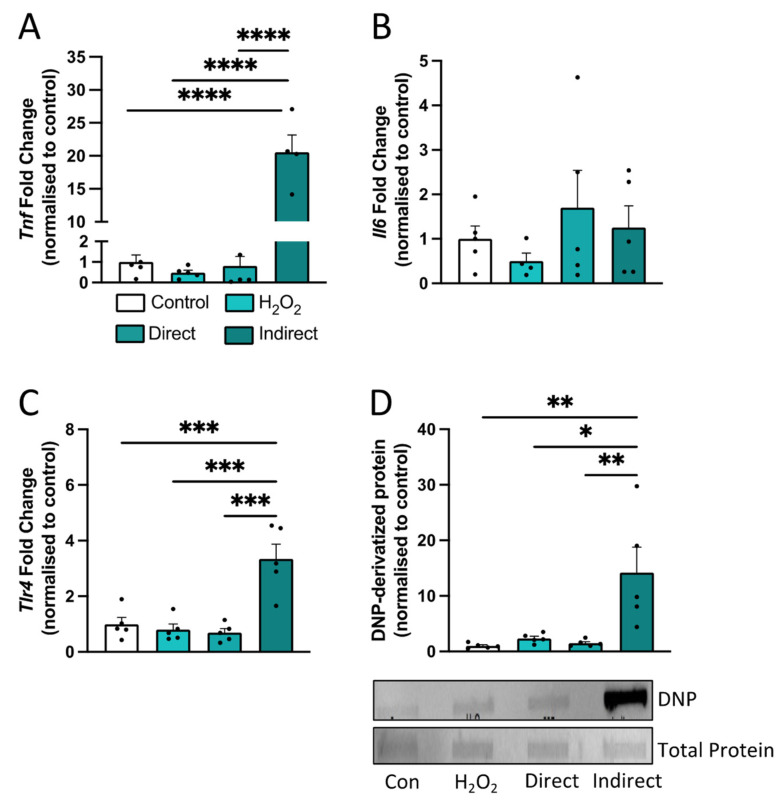
Indirect cigarette smoke extract (CSE) conditioned medium evoked a pro-inflammatory and oxidative stress response in human microglial cells (HMC-3). Lung epithelial cells (BEAS-2B) were exposed to cigarette smoke extract (CSE [25%]) for 6 h (indirect), and culture medium was transferred onto HMC-3 cells and incubated for 24 h (n = 6 per group). HMC-3 cells were also either exposed to 10% CSE (direct) or hydrogen peroxide (H_2_O_2_) for 24 h (n = 6 per group). (**A**–**C**) Gene expression of inflammatory markers in HMC-3 cells, including (**A**) tumor necrosis factor-alpha (*Tnfα*)*,* (**B**) interleukin-6 (*Il6*), and (**C**) toll-like receptor 4 (*Tlr4*). (**D**) DNP-derivatized protein in HMC-3 cells. Data are expressed as mean + SEM. * *p* < 0.05, ** *p* < 0.01, *** *p* < 0.001, **** *p* < 0.0001.

We hypothesized that inflammatory and oxidative stress mediators from CS-exposed lungs ‘spill-over’ into the systemic circulation, leading to extra-pulmonary comorbidities and activation of microglia. To ascertain the contributory role of airway inflammation on microglial signaling, we transferred culture medium from BEAS-2B cells, which were exposed to CSE (i.e., indirect CM), onto HMC-3 cells. Microglial cells were also either exposed to 10% CSE or H_2_O_2_. We found that exposure to indirect CM for 24 h resulted in a marked increase in the expression of *Tnfα* (*F*_(3,15)_ = 68.34; *p* < 0.0001; Figure 10A) and *Tlr4* (*F*_(3,16)_ = 15.76; *p* < 0.0001; Figure 10C) but not *Il6* (Figure 10B) following incubation with indirect CM, while exposure to 10% CSE or H_2_O_2_ did not alter pro-inflammatory gene expression. We next examined the oxidative stress profiles, and we found that indirect CSE exposure induced a significant increase in protein carbonylation (*F*_(3,16)_ = 7.670; *p =* 0.0023; Figure 10D) in HMC3 cells when compared to control media. Collectively, the results suggest that exposure to indirect CSE containing pro-inflammatory mediators from lung epithelial cells, but not direct CSE exposure, evoked a pro-inflammatory and oxidative stress response in HMC-3 cells.

**Figure 11:**
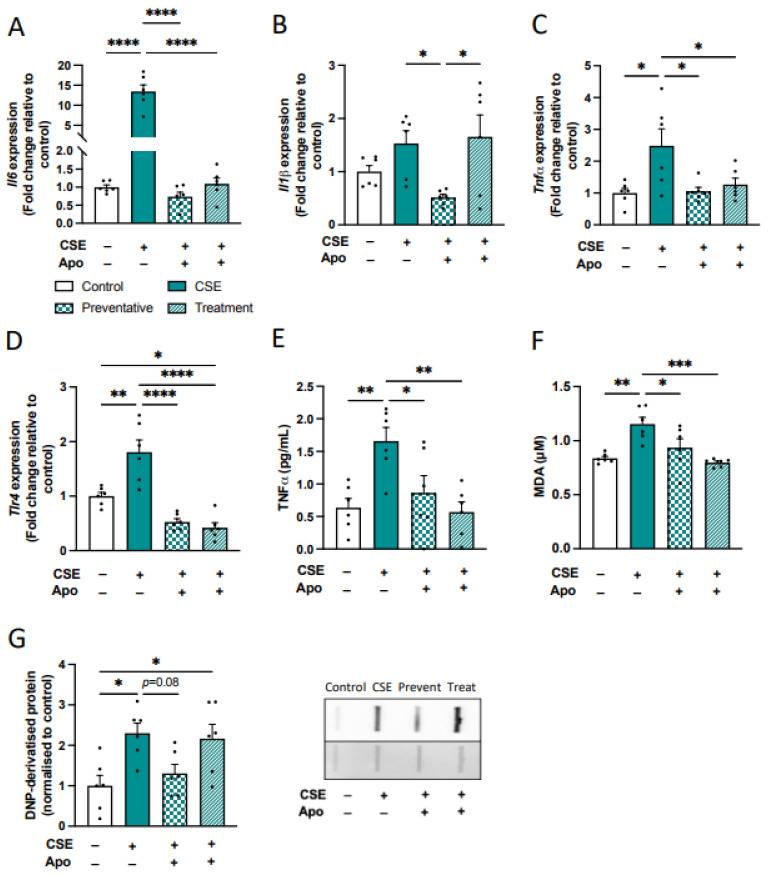
Treatment with apocynin prevented cigarette smoke extract (CSE)-induced inflammation and oxidative stress in human microglial cells (HMC-3). HMC3 cells were treated with CSE-indirect condition medium derived from BEAS-2B cells treated with apocynin (500 nM) as co-administration (preventative) or therapeutic for 24 h. (**A**–**D**) Gene expression of inflammatory markers in HMC-3 cells. (**A**) interleukin 6 (*Il6*). (**B**) *Il1β.* (**C**) tumor necrosis factor-alpha (*Tnfα*). (**D**) Toll-like receptor 4 (*Tlr4*; n = 6 per group). (**E**) TNF cytokine and(**F**) MDA levels in the supernatant measured by ELISA and the TBARS assay kit, respectively (n = 6 per group). (**G**) DNP-derivatized protein in HMC-3 cells. Data are expressed as mean + SEM. * *p* < 0.05, ** *p* < 0.01, *** *p* < 0.001, **** *p* < 0.0001.

To determine whether microglial cells are the key contributor to the elevated pro-inflammatory and oxidative stress profiles following CS exposure in our preclinical model, we next examined whether targeting oxidative stress, using apocynin, could attenuate the pro-inflammatory profile of HMC-3 cells following indirect CSE exposure. HMC3 cells were treated with indirect CM derived from BEAS-2B cells treated with apocynin (500 nM) as co-administration (preventative) or therapeutic for 24 h. Apocynin, when administered prophylactically and therapeutically, significantly downregulated the expression of pro-inflammatory cytokines *Il6, Tnfα,* and *Tlr4,* whereas *Il1β* expression was significantly reduced following only the preventative apocynin treatment (*Il6*: *F*_(3,20)_ = 56.62, *p* < 0.0001; Figure 11A, *Il1b*: *F*_(3,20)_ = 4.479, *p* = 0.01; Figure 11B, *Tnfα*: *F*_(3,20)_ = 5.411, *p* = 0.0068; Figure 11C, *Tlr4*: *F*_(3,20)_ = 24.05, *p* < 0.0001; Figure 11D). In accordance with gene expression, the level of Tnfα in the supernatant was significantly reduced by apocynin both as preventative and therapeutic (*F*_(3,20)_ = 6.401, *p* = 0.0032; Figure 11E). Apocynin treatment both as co-administration and therapy significantly decreased the level of lipid peroxidation in HMC3 stimulated with indirect CM, while the level of carbonylated protein was decreased following the preventative CM treatment only (MDA: *F*_(3,20)_ = 9.154, *p* = 0.0005; Figure 11F, DNP-derivatized protein: *F*_(3,20)_ = 5.343, *p* = 0.0072; Figure 11G). Taken together, the results indicate that indirect CM containing the inflammatory mediators secreted by lung epithelial cells exposed to CSE elicits an augmented pro-inflammatory and oxidative stress response in HMC3 cells, confirming that the inflammatory response of lung cells following CS exposure may indeed “spill over” into the CNS, leading to the disruption of microglial cells.

### 3.9. Neuroprotective Effects of Apocynin Are Mediated by Inhibition of the JNK Signaling Pathway

**Figure 12:**
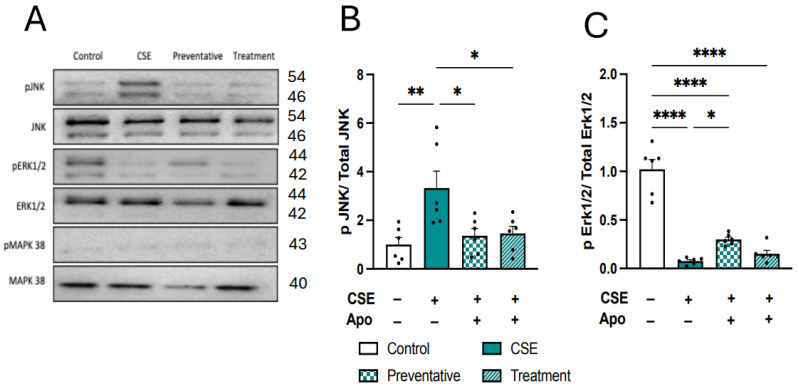
Apocynin altered the expression of mitogen-activated protein kinases (MAPK) signaling pathways in HMC-3 cells following exposure to cigarette smoke extract conditioned media from BEAS-2B cells. (**A**) Representative image of protein expression of phosphorylated and total c-Jun N-terminal kinase (JNK), extracellular signal-regulated kinase (ERK1/2), and p38 in HMC3 cells. (**B**) Phospho-JNK; (**C**) Phospho-ERK1/2, (n = 6 per group). Data are expressed as mean + SEM. * *p* < 0.05, ** *p* < 0.01, **** *p* < 0.0001.

To understand how apocynin treatment improves microglial pro-inflammatory and oxidative stress pathways, we assessed the mitogen-activated protein kinases (MAPKs) signaling pathway. MAPK has been implicated in several CNS processes, such as memory and the response to antidepressants [59], and plays a pivotal role in the production of pro-inflammatory cytokines and the subsequent events that lead to inflammation [60]. Indirect CM elicited rapid phosphorylation of c-Jun N-terminal kinase (pJNK; *F*_(3,20)_ = 5.871, *p* = 0.0048, Figure 12A,B) while inhibiting the phosphorylation of extracellular signal-regulated kinase (pERK1/2; *F*_(3,20)_ = 62.70, *p* < 0.0001, Figure 12A,C). p-38 was not changed in response to indirect CM (Figure 12A). Increased phosphorylation of JNK was significantly inhibited by co-administration and treatment with apocynin (*F*_(3,20)_ = 5.871, *p* = 0.0048, Figure 12A,B). Whereas inhibited ERK1/2 phosphorylation (pERK1/2) was partially restored with co-administration of apocynin (*F*_(3,20)_ = 62.70, *p* < 0.0001, Figure 12A,C). Taken together, CM from BEAS-2B cells exposed to CSE alters microglial inflammatory and oxidative stress profiles, and this may be via a MAPK-dependent signaling pathway. Importantly, treating BEAS-2B epithelial cells with the NOX2 inhibitor, apocynin, can improve the altered microglial profile driven by indirect CM.

## 4. Discussion

Neurocognitive impairments, including learning and memory deficits and anxiety, are common comorbidities among people with COPD and are often worsened during or following episodes of acute exacerbations. This study examined the mechanisms underlying CS-induced neurocognitive impairments and whether inhibiting inflammation and oxidative stress using the NOX2 inhibitor apocynin could prevent these impairments. Our results showed that excessive lung inflammation was associated with both anxiety-like behaviors and impairments in recognition memory. This was associated with increased microglial activation and altered neuronal profiles. Apocynin treatment was effective in alleviating systemic inflammation and lipid peroxidation, as well as anxiety-like behaviors, while preserving normal microglial morphology in the amygdala and hippocampus. However, it was unable to restore recognition memory impairments or the altered hippocampal neuronal profile. The neuroprotective effect of apocynin may be attributed to alterations in the MAPK signaling pathway.

In the lungs, CS exposure causes an abnormal inflammatory response, which may promote airway remodeling and parenchymal destruction, leading to the manifestation of COPD [61,62]. Neutrophils are key players in the pathogenesis of COPD, and several clinical studies have reported a marked increase in neutrophils in the BALF and sputum of COPD patients [63,64]. Moreover, neutrophils are a critical source of ROS, secreting a number of proteases that contribute to lung destruction and the development of emphysema [63,64]. In the present study, we demonstrated that the NOX2 inhibitor, apocynin, was able to reduce CS-induced BALF inflammation, as evidenced by a reduction in neutrophils. This is in line with previous preclinical studies showing that prophylactic apocynin administration can improve lung inflammation by attenuating neutrophil infiltration into the lungs [10,56]. Conversely, Boshtam and colleagues have demonstrated that apocynin may act by attenuating pro-inflammatory mediators [65], although we found no significant change in the expression of CS-induced inflammatory cytokines following a prophylactic treatment with apocynin. Although gene expression was only assessed at a single timepoint, this does suggest that other immune regulatory cells may be involved. For example, alveolar macrophages secrete inflammatory mediators and proteases when activated by CS and other irritants [66]. Hence, it is possible that the persistent increase in macrophages may be responsible for the perpetuation of the inflammatory response in the lungs.

Beyond lung inflammation, mice exposed to CS have widespread inflammation throughout the body (elevated systemic CRP) and an imbalance between antioxidants and free radicals (i.e., oxidative stress). Clinical studies have shown elevated levels of inflammatory and oxidative stress markers in the blood of people with COPD, including CRP [67,68,69,70], TNFα [71], IL6 [67], IL8 [70], and MDA [68]. Exhaled breath analysis also revealed elevated levels of H_2_O_2_ in current smokers and people with COPD when compared to non-smokers [72,73]. Notably, while smoking itself can trigger systemic inflammation, the degree of inflammation in smokers with COPD is significantly higher than in current smokers without COPD [74,75]. COPD is thought to cause chronic low-grade inflammation and oxidative stress throughout the body due to the ‘spill-over’ from the CS-exposed lungs. This is believed to be a major contributor to extra-pulmonary comorbidities in people with COPD [7]. Our study uniquely showed that apocynin, delivered daily through injections into the peritoneal cavity (i.p. injection), completely prevented CS-induced systemic inflammation and oxidative stress in the blood of mice. Similar benefits were shown in other murine models. For instance, Rahman and colleagues showed that apocynin caused a reduction in serum MDA in carbon tetrachloride-induced liver damage in rats [76]. Moreover, Kouki et al. recently reported that oral supplementation of rats with apocynin reduced several systemic inflammatory markers, including CRP, in an ulcerative colitis model [77].

In the general population, anxiety and depression have been implicated in the risk of cognitive decline [78]. For instance, there is up to a 45% greater risk of cognitive impairment in people with anxiety disorders [79]. Lindert et al. recently demonstrated that anxiety symptoms were associated with a decline in episodic memory and executive function in both men and women, potentially due to the overactivation of the hypothalamic-pituitary-adrenal (HPA) axis causing immune dysregulation leading to damage to the hippocampus and subsequent changes in cognition [79]. Moreover, there is an association between depression and anxiety disorders and cognitive impairment in people with COPD [13,80,81]. We have demonstrated that CS-exposed mice displayed a neophobic phenotype, and this was associated with impaired social recognition memory in the social interaction task [3]. In support of our finding, apocynin has been shown to have an anxiolytic effect in numerous models of anxiety and depression [82,83,84]. Despite improvements in anxiety-like behaviors, we were unable to prevent recognition memory impairments with apocynin in our CS-exposed mice. Our finding aligns with results from the human nocturnal oxygen therapy (NOTT) trial, which showed that modest improvement in cognitive function was not associated with improvement in emotion or depressive symptoms [85]. This suggests that in the present study, recognition memory impairments may be independent of inhibition of NOX2 activation.

Alongside the CS-induced anxiogenic effects, we observed an increase in locomotor activity in CS-exposed mice, suggesting increased exploratory behaviors. One possible explanation for increased exploration may be attributed to the effect of nicotine. Liu and colleagues [86] reported that subcutaneous administration of nicotine induces hyperlocomotion in the OF task in mice. Similarly, Pelgrim et al. illustrated that CS-exposed mice displayed increased locomotor activity and spent more time in the center of the arena, irrespective of lipopolysaccharide (LPS) exposure [87], suggesting that nicotine is likely to be implicated in their altered behavior [87]. Remarkably, we found that the observed increased locomotion was suppressed in a novel object recognition task. However, it is noteworthy that the OF task was performed before the novel recognition test; therefore, it is likely that the habituation due to multiple exposures to the arena may result in decreased locomotion in the novel recognition test [87]. While some cognitive changes may be attributed to nicotine, there are studies that have shown that nicotine-free e-cigarettes have impaired novel object recognition performance [88] and impaired cognition in mice exposed to e-cigarettes independent of nicotine [89]. Nicotine has been known to enhance cognition rather than deteriorate [90]. Hence, the impaired cognition seen in our study may be happening due to mechanisms independent of nicotine.

Recent research suggests that neuroinflammation is emerging as a key modulator of neurological disturbances, including both anxiety disorders and cognitive function [91,92,93]. During acute stress, the resident immune cells, specifically microglia and astrocytes, become activated and release inflammatory and oxidative stress mediators. However, chronic dysregulation of microglial cells may result in the accumulation of inflammatory mediators, leading to neurocognitive impairments [94]. Given that intact neurocognitive function is associated with functional connectivity between multiple anatomical brain regions [95,96,97,98], this study is the first to investigate the relationship between neurocognitive function and microglia in the amygdala and hippocampus following CS exposure. We demonstrate that mice exposed to CS displayed heightened anxiety-like behaviors and recognition memory deficits, and this was associated with reactive microglial profiles in the amygdala and hippocampus. It is important to note that, while the hippocampus is mainly involved in cognitive functioning, it has been implicated alongside the amygdala in modulating anxiety phenotypes [99]. This aligns with our previous findings that CS-induced working and social recognition memory is linked to microglial activation within the hippocampus [53] and in the hypothalamus following chronic CS exposure for 24 weeks [3]. In accordance with the current study, it was reported that CS-induced hyperlocomotion was associated with increased microglial activation in the hippocampus of mice chronically exposed to CS and LPS for 72 days [87]. Notably, pharmacologically depleting microglia (using the selective colony-stimulating factor-1 receptor [CSF1R] inhibitor, PLX5622) in acute withdrawal mouse models prevented withdrawal-induced anxiety-like behaviors [100,101]. These findings support our hypothesis that CS exposure activates microglia within crucial anatomical brain regions and induces anxiety-like behaviors.

Neurocognitive dysfunction, including anxiety disorders and impaired learning and memory, has been associated with an imbalance of oxidative stress and antioxidants [102,103]. Microglial cells are a major source of oxidative stress and we have demonstrated that inhibiting NOX2 activation via apocynin partially restored the microglial profile within the amygdala and hippocampus as well as the pro-inflammatory gene expression in the amygdala following 8 weeks of CS exposure. Several reports demonstrated that targeting microglial activation using other pharmacological treatments such as minocycline promoted improvements in depression and anxiety-like behaviors in chronic stress models and after brain ischemia [104,105,106]. Despite this, there is limited literature on the effect of apocynin or other antioxidants on microglial activation in preclinical models of COPD. De Luca and colleagues have also shown that ebselen, the antioxidant GPX1 mimetic, was able to completely prevent recognition memory impairments in CS-exposed mice but was unable to restore microglial profiles [53]. However, similar to the current study, we have recently shown that a prophylactic apocynin treatment was able to prevent social recognition memory impairments but not neophobia, alongside a complete prevention of microglial activation in the suprachiasmatic nucleus of the hypothalamus [3], thus suggesting that whilst antioxidant treatment may improve recognition memory, inhibition of NOX2 activity is crucial to improving both microglial profiles and anxiety-like behaviors in CS-exposed mice but not recognition memory.

Eight weeks of CS exposure resulted in a significantly elevated number of post-mitotic DCX-positive cells in the SG region of the dentate gyrus, which may indicate enhanced neurogenesis. CS exposure also triggered a concurrent suppression of synaptophysin, a presynaptic marker, compared to sham mice. Based on our current findings that there is a significant restoration of microglial profiles within both the hippocampus and amygdala following co-administration of CS and apocynin, we hypothesized that co-administration of CS and apocynin would normalize both neuronal and synaptic profiles within the hippocampus. While apocynin effectively prevented the CS-mediated increase in post-mitotic DCX-positive cells, it failed to restore synaptophysin levels or alleviate recognition memory impairments in CS-exposed mice. This aligns with preclinical studies in Alzheimer’s disease, which revealed that apocynin treatment, while reducing the oxidative stress burden within the brain, failed to improve spatial learning and memory, or hippocampal synaptic deficits [107,108]. Interestingly, a recent study by Zheng and colleagues demonstrated that LPS-induced microglial activation and anxiety-like behaviors were associated with alterations in BLA neuronal plasticity. Specifically, they observed an increase in excitatory presynaptic glutamate transmission, suggesting a shift in the excitatory/inhibitory balance [34]. While the current study did not characterize the specific presynaptic alterations in CS-exposed mice, we observed a reduction in presynaptic density alongside an activated microglial morphology, thus suggesting potential dysregulation of the excitatory/inhibitory balance. Future investigations are warranted to assess the balance between excitatory and inhibitory presynaptic transmissions following CS exposure and their potential contribution to recognition memory impairments.

The question remains: how does lung inflammation following CS exposure cause microglial activation? In microglia, the MAPK signaling pathway is critical in modulating inflammatory responses to stressors [60]. In the present study, we found that the indirect CSE-induced phosphorylated JNK was inhibited by treating BEAS-2B lung epithelial cells with apocynin. Increased activation of JNK is known to promote the overexpression of pro-inflammatory factors such as TNFα and IL1β [109]. Moreover, JNK signaling regulates neuronal death and plays a crucial role in brain development and synaptic plasticity [110], and hyperactivation of the JNK pathway has been implicated in the pathogenesis of neurodegenerative diseases such as Alzheimer’s disease [110,111]. While inhibition of JNK ameliorated neuroinflammation-induced depressive-like behavior [112], another MAPK protein, ERK, plays a critical role in various neuronal processes, including survival, differentiation, synaptic plasticity, and memory formation [113]. We demonstrate that treating lung epithelial cells exposed to CSE with apocynin enhanced the expression of phosphorylated ERK1/2 (pERK1/2) in microglial cells. It has been suggested that inhibition of ERK-dependent activation of NADPH is critical for neuroprotective potential mediated by apocynin [114]. For instance, Dang *et al.* demonstrated that apocynin protected against oxidative damage, microglial activation, and pro-apoptotic signaling via inhibition of methamphetamine-induced ERK phosphorylation and subsequent activation of p47phox [115]. Conversely, other studies have suggested that ERK1/2 phosphorylation plays a key role in the regulation of nNOS expression as well as hippocampal neurogenesis and synaptogenesis of newborn neuron [116]. In the same context, our studies are consistent with those conducted by Ibrahim et al. [113] who report that diapocynin, a dimer of apocynin, attenuates memory deficits and Alzheimer’s disease-like anomalies via activation of ERK while inhibiting the JNK/cJun signaling pathway. Hence, treating lung epithelial cells with apocynin may potentially reduce the production of cytokines and chemokines within the indirect CM, thus suppressing the activation of ERK1/2 as well as inhibiting the JNK pathway in microglial cells. Therefore, apocynin may represent a novel way to treat neuroinflammation and potentially improve anxiety disorders.

It is noteworthy that clinical studies have shown that there are gender differences in the prevalence of neurocognitive comorbidities in people with COPD [117]. Compared to men, women with COPD experience higher levels of anxiety symptoms [118], and it is estimated that about 56% of anxiety disorders occur in females with COPD compared to 35% in males [119]. This sex disparity may be linked to the potentially higher lifetime prevalence of mood disorders in women compared to men [119]. While some studies suggest that cognitive disorders and dementia are more prevalent among males with COPD [117], other reports show no significant difference between males and females with COPD [26]. A limitation of the current study is that the data were obtained exclusively from male mice. To address this gap in knowledge, future studies should incorporate both male and female mice as well as ovariectomized female mice. Approximately 10% of COPD patients suffer from coexisting depression and anxiety disorders [16]. Moreover, mouse models of anxiety-like behaviors may also display depressive-like behaviors, reflecting the high comorbidity between anxiety disorders and depression in humans [120]. Anhedonia and other depressive-like behaviors were not explored in our model of COPD, therefore, further research including more extensive behavior paradigms to assess the depressive-like behavior is needed.

**Figure 13:**
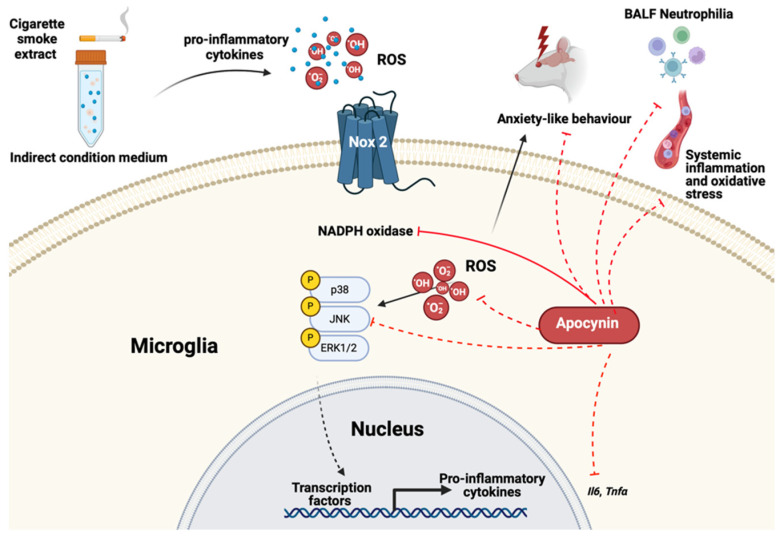
Potential mechanism of action of apocynin in vivo and in vitro following cigarette smoke exposure. We demonstrated that targeting cigarette smoke-induced lung inflammation and oxidative stress using concomitant apocynin treatment alleviated systemic inflammation and oxidative stress as well as anxiety-like behaviors in BALB/c mice. Stimulating human microglial cells (HMC-3) with a conditioned media treatment derived from lung epithelial cells treated with cigarette smoke extract led to upregulation of the expression of pro-inflammatory cytokines and oxidative stress markers. Apocynin reduced pro-inflammatory cytokine expression, potentially through inhibition of the c-Jun N-terminal kinase (JNK) signaling pathway. BALF: bronchioalveolar lavage fluid, ROS: reactive oxygen species, ERK1/2: extracellular signal-regulated kinase 1 and 2, Nox2: NADPH oxidase 2, Il-6: interlukin-6, Tnfα: tumor necrosis factor-alpha.

In summary, our work provides evidence for the interplay between lung and systemic inflammation and oxidative stress and its contribution to neurocognitive dysfunction in COPD (Figure 13). A novel finding in our study was that targeting oxidative stress using apocynin augmented systemic complications and alleviated anxiety-like behavior and microglial activation in the amygdala and hippocampus. However, apocynin was unable to prevent recognition and memory impairment alongside neurogenesis and synaptogenesis. This finding implies that recognition memory may be independent of oxidative stress and inflammation, whereas other aspects of neurocognition, such as anxiety-like behavior, are acquiescent to the effects of antioxidant mediators. Our results contribute to the development and utilization of antioxidants as a novel interventional strategy to treat neurocognitive comorbidities in people with COPD.

## Figures and Tables

**Table 1:** TaqMan assay details (Life Technologies, Carlsbad, CA, USA) used for qRT-PCR.

Target Gene	Gene Accession Number	NCBI Reference Sequence	TaqMan Assay ID	Amplicon Size (bp)
*Gapdh*	NM_001289726	NM_002046.3	Mm99999915_g1	133
*Rps18*	NM_011296	NM_011296.2	Mm02601777-g1	76
*Il6*	NM_031168	NM_031168.1	Mm00446190_m1	78
*Il1* *β*	NM_008361	NM_008361.3	Mm00434228_m1	90
*Tnf* *α*	NM_001278601	NM_013693.3	Mm00443258_m1	81
*Ccl2*	NM_011333	NM_ 011333.3	Mm00441242_m1	74
*Cxcl2*	NM_009140	NM_009140.2	Mm00436450_m1	67
*Cybb (Nox2)*	NM_007807	NM_007807.5	Mm01287743_m1	63
*Nos2*	NM_010927	NM_010927.3	Mm00440502_m1	66
*Gpx1*	NM_008160	NM_008160.6	Mm00656767_g1	134
*Il6*	NM_000600	NM_000600.4	Hs00174131_m1	95
*Il1* *β*	NM_000576	NM_000576.2	Hs01555410_m1	91
*Tnf* *α*	NM_000594	NM_000594.3	Hs00174128_m1	80
*Tlr4*	NM_003266	NM_003266.3	Hs00152939_m1	89

**Table 2:** Number of Iba-1-positive cells in amygdala nuclei.

Region	Sham Vehicle	CS Vehicle	Sham Apocynin	CS Apocynin
**LA**	18.88 ± 0.690	13.69 ± 0.643 ***	17.94 ± 0.745	15.50 ± 1.002
**BLA**	45.46 ± 1.839	38.05 ± 1.128	43.479 ± 2.823	36.56 ± 1.718
**BMA**	20.42 ± 0.610	17.52 ± 1.115	19.77 ± 0.673	18.67 ± 0.742
**CeA**	8.93 ± 0.102	7.50 ± 0.352 *	9.33 ± 0.418	9.29 ± 0.267 **

* *p* < 0.05, ** *p* < 0.001, *** *p* < 0.001.

**Table 3:** Number of Iba-1-positive cells in hippocampus subregions.

Region	Sham Vehicle	CS Vehicle	Sham Apocynin	CS Apocynin
**CA1**	26.75 ± 1.288	24.82 ± 0.977	23.36 ± 1.076	29.13 ± 1.33 **
**CA3**	39.08 ± 1.536	38.57 ± 2.860	34.93 ± 1.848	34.80 ± 1.432
**SG**	17.42 ± 0.468	15.50 ± 1.292	15.62 ± 0.936	14.67 ± 1.011
**Molecular**	19.50 ± 0.604	17.63 ± 1.372 *	21.85 ± 1.325	17.20 ± 0.975 *
**Hilus**	6.93 ± 0.263	6.40 ± 0.496	5.98 ± 0.838	7.03 ± 0.280

* *p* < 0.05; ** *p* < 0.001.

## Data Availability

The datasets supporting the conclusions of this article are available upon request.
